# Mild Mitochondrial Uncoupling for True Ectopic Lipid Disposal

**DOI:** 10.3390/ijms26167740

**Published:** 2025-08-11

**Authors:** Hui-Young Lee

**Affiliations:** 1 Laboratory of Mitochondria and Metabolic Diseases, Lee Gil Ya Cancer and Diabetes Institute, Gachon University, Incheon 21999, Republic of Korea; hylee@gachon.ac.kr; Tel.: +82-32-899-6236; 2 Department of Health Sciences and Technology, GAIHST, Gachon University, Incheon 21999, Republic of Korea; 3 Department of Molecular Medicine, College of Medicine, Gachon University, Incheon 21936, Republic of Korea

**Keywords:** ectopic lipids, ballooning effects, mitochondrial uncoupling, metabolic diseases

## Abstract

Ectopic lipid accumulation is a core contributor to insulin resistance and metabolic diseases, including type 2 diabetes, dyslipidemia, and non-alcoholic fatty liver disease. Conventional therapies have primarily focused on redistributing lipid burden across tissues or modulating specific pathways. However, this often causes compensatory responses that merely shift the burden rather than resolve the underlying lipid excess. In this review, we introduce the concept of the ballooning effect, wherein single-target interventions inadvertently exacerbate lipid accumulation in non-target tissues. We then explore fundamental strategies for true lipid disposal, which aim either to prevent lipid influx or to promote complete lipid oxidation. Among these, mild mitochondrial uncoupling emerges as a promising solution. By dissipating substrate energy as heat, mitochondrial uncoupling reduces ectopic lipid burden without relying on redistribution. Recent advances have yielded safer chemical uncouplers and novel endogenous protein-based mechanisms that enable controlled uncoupling with minimal toxicity. Together, these provide a new framework for next-generation metabolic therapies that move beyond lipid redistribution and aim for a true lipid disposal, potentially offering a safe and effective strategy.

## 1. Introduction

Metabolic diseases—including type 2 diabetes, dyslipidemia, non-alcoholic fatty liver disease (NAFLD), and obesity—are increasing globally and share a central pathogenic mechanism: the accumulation of excess fat in non-adipose tissues, resulting in insulin resistance and organ dysfunction [1,2,3]. Shulman and colleagues, using advanced magnetic resonance spectroscopy and tracer studies, demonstrated that ectopic lipid accumulation in the skeletal muscle and liver is a primary driver of whole body insulin resistance and the metabolic syndrome [2,4,5,6]. Importantly, this phenomenon is not limited to obese individuals, as recent studies have shown that even non-obese Asians, who often have a relatively low fat mass, are susceptible to metabolic abnormalities due to ectopic fat accumulation in the liver and skeletal muscle [7]. This further elaborate that intracellular lipid accumulation in ectopic tissues impairs insulin signaling and promotes a cascade of metabolic disturbances, including type 2 diabetes and dyslipidemia, elucidating the harmful relationship between ectopic lipid deposition and insulin resistance across multiple organs [3].

**Figure 1:**
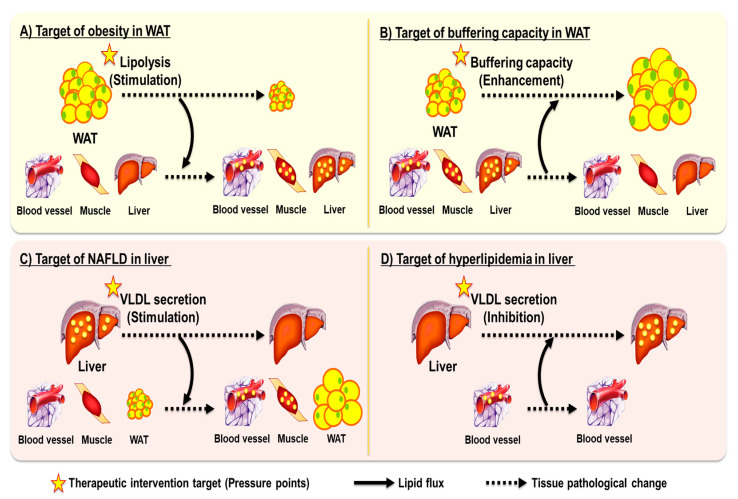
Conceptual representation of the “ballooning effect” in metabolic disease treatment. This schematic illustrates the core concept of the ballooning effect, where targeting a single metabolic tissue or pathway (indicated as yellow pressure points) often leads to unintended redistribution of metabolic burden, resulting in new pathological states in other organs. (**A**) Stimulating lipolysis in WAT to treat obesity reduces fat mass, but elevates circulating FFAs and glycerol, which are taken up by liver and muscle, leading to ectopic lipid accumulation and insulin resistance. (**B**) Enhancing buffering capacity in WAT can mitigate steatosis and hyperlipidemia by trapping lipids within adipose tissue, but results in WAT expansion, lead to obesity. (**C**) Stimulating hepatic VLDL secretion improves hepatic steatosis but increases plasma triglycerides and cholesterol, which are redistributed to WAT and muscle. (**D**) Inhibiting VLDL secretion reduces plasma lipids but exacerbates hepatic lipid accumulation and NAFLD. WAT, white adipose tissue; FFAs, free fatty acids; VLDL, very-low-density lipoprotein; NAFLD, non-alcoholic fatty liver disease; TG, triglyceride.

However, therapeutic strategies aimed at reducing lipid accumulation in a single organ often lead to compensatory lipid deposition in other tissues—a phenomenon we refer to as the “ballooning effect” in the context of therapeutic interventions for lipid-mediated metabolic diseases (Figure 1). Much like pressing one side of a balloon causes expansion on another, targeting adipose tissue lipolysis or hepatic lipid secretion in isolation may not reduce the overall ectopic lipid pool but merely shift its distribution. This redistribution, rather than resolution, of excess lipids highlights a critical limitation of compartment-specific interventions. Importantly, such compensatory lipid redistribution is particularly problematic under sedentary conditions, where systemic energy demand is low. In contrast, during physiologic states of high energy expenditure—such as exercise or cold exposure—increased lipolysis is counterbalanced by enhanced lipid oxidation, reducing the risk of ectopic lipid accumulation [8,9].

This underscores the need for either integrated, multi-organ therapeutic approaches or fundamental strategies that enable true lipid disposal at the systemic level. In this review, we first introduce the concept of ballooning effects as a therapy-induced, maladaptive redistribution of lipids across metabolic organs. We then examine prior attempts at true lipid disposal, including their mechanisms and limitations. Lastly, we explore mild mitochondrial uncoupling as a potentially safer and more effective approach to promoting true lipid disposal, with particular attention to emerging targets beyond classical uncoupling proteins (UCPs). Together, these emerging strategies may represent a novel paradigm for true lipid disposal, capable of mitigating the ballooning effects caused by lipid redistribution and overcoming the inherent limitations of conventional compartment-specific interventions.

## 2. Pathophysiological Role of Ectopic Lipids in Metabolic Disorders

### 2.1. Physiological Routes of Dietary Lipid Processing

Dietary lipids are processed through a tightly regulated sequence of digestion, absorption, transport, and tissue distribution. Most digestion occurs in the small intestine, where pancreatic lipase hydrolyzes triglycerides into fatty acids and monoglycerides [10,11]. These products form micelles with bile salts, which facilitate their transport across the unstirred water layer to the brush border of enterocytes [12]. Inside enterocytes, fatty acids are re-esterified into triglycerides and packaged with cholesterol, phospholipids, and apolipoprotein B (ApoB) 48 into nascent chylomicrons [10]. These chylomicrons are secreted into intestinal lymphatics and delivered to the bloodstream via the thoracic duct [13]. Chylomicron remnants are taken up by the liver where their lipid components are repackaged into very low-density lipoproteins (VLDL) containing ApoB100 [10]. The liver also converts excess carbohydrates to fat through de novo lipogenesis and incorporated it into the VLDL [2,10]. Then, VLDL circulate and are hydrolyzed by lipoprotein lipase on capillary endothelium, releasing fatty acids for uptake into peripheral tissues including skeletal muscle and adipose tissue, and this fatty acids are oxidized for energy in muscle or stored as triglycerides in adipose tissue [2,10]. In this context, adipose tissue functions as the primary and safest lipid reservoir under normal physiological conditions.

### 2.2. Lipid Spillover to Non-Adipose Tissues

If excess dietary fat is stored exclusively in adipose tissue, the system remains metabolically buffered, often resulting in obesity but not overt metabolic disease. This phenomenon is observed in some individuals with metabolically healthy obesity, who maintain relatively low hepatic lipid content despite increased adiposity [14]. However, in the context of sustained overnutrition and low physical activity—typical of modern sedentary lifestyles—adipose tissue can reach its storage threshold or become functionally impaired. In such cases, lipids begin to accumulate in non-adipose tissues such as liver, skeletal muscle, and pancreas, a process known as ectopic lipid accumulation [2]. This phenomenon maybe reflects an evolutionarily conserved energy redistribution mechanism that once provided survival advantages. In ancestral environments marked by intermittent food availability and predators, the body evolved flexible pathways to buffer energy fluctuations from starvation or fleeing. Rapid lipid redistribution to the liver during fasting [15] or exercise [16] may ensure energy availability without delay, offering survival advantages under certain conditions.

However, in today’s environment of persistent caloric excess without proportional energy expenditure, the same redistribution mechanism becomes maladaptive. Critically, insulin resistance, a key precursor to metabolic diseases like type 2 diabetes, arises when the body’s energy storage systems are exposed to a chronic energy surplus, leads to a critical initial event: ectopic lipid accumulation [2]. The ectopic lipid accumulation impairs insulin signaling within insulin-sensitive tissues, such as skeletal muscle and liver [2]. This impairment has distinct consequences: in skeletal muscle, it leads to reduced glucose uptake, while in the liver, it results in decreased hepatic glycogen synthesis [2]. Importantly, muscle insulin resistance typically precedes liver insulin resistance, setting the stage for systemic metabolic deterioration [2]. Recent clinical studies further support the organ-specific hierarchy of ectopic lipid deposition [17,18]. Quantitative imaging analysis reveals that skeletal muscle accumulates lipids most rapidly, followed by the pancreas and liver in diabetes patients compared to non-diabetic subjects [17]. In individuals with obesity, ectopic fat content is notably high in muscle [18] and interestingly in pancreas [17], underscoring their central role in tissue-specific insulin resistance and beta-cell dysfunction.

### 2.3. Ectopic Lipid Accumulations in Skeletal Muscle, Liver and Pancreas

Skeletal muscle insulin resistance plays a central role in the early pathogenesis of metabolic disorders. Impaired insulin signaling in muscle significantly reduces glucose uptake and glycogen synthesis, rerouting postprandial carbohydrate flux toward the liver [2,6,19]. This shift accelerates hepatic de novo lipogenesis (DNL), leading to increased triglyceride synthesis [2,6]. Petersen et al. demonstrated that this metabolic rerouting occurs independently of visceral fat or circulating adipokines, suggesting that muscle insulin resistance precedes hepatic metabolic dysfunction [6]. In parallel, excessive lipolysis from dysfunctional or saturated adipose tissue—often observed in obesity or during therapeutic fat reduction—contributes additional free fatty acids (FFAs) to the liver and skeletal muscle. Under sedentary or low energy demand conditions, these FFAs are not efficiently oxidized, instead accumulating as ectopic lipids within insulin-sensitive tissues [20,21]. This lipid overflow exacerbates lipotoxicity, promoting intracellular accumulation of diacylglycerols and ceramides, which activate stress kinases and impair insulin receptor signaling pathways [2,3,4]. In the liver, this not only worsens steatosis but also promotes hepatic insulin resistance, gluconeogenesis, and dyslipidemia. Hepatic DNL and FFA uptake synergistically overload VLDL export mechanisms, further elevating circulating triglycerides and contributing to the development of NAFLD [2,6,22]. Thus, two parallel but interlinked mechanisms, (1) carbohydrate overflow due to skeletal muscle insulin resistance, and (2) FFA spillover from adipose tissue lipolysis, converge to drive ectopic lipid accumulation in the liver and muscle. Even under beneficial conditions such as exercise, if FFA oxidation capacity is exceeded, lipid deposition may still occur [9]. These processes reinforce the interconnected nature of lipid metabolism and its dysregulation in obesity, insulin resistance, NAFLD, and other metabolic diseases.

Although skeletal muscle and liver are the primary sites of ectopic lipid accumulation and insulin resistance, emerging evidence suggests that the pancreas is also vulnerable to lipid overload. Pancreatic steatosis has been increasingly recognized as more than a bystander condition [23,24,25]. Recent studies show that its impact on insulin secretion may vary depending on individual genetic predisposition and ethnicity [23,25]. While glucose remains the dominant regulator of β-cell activity, chronic exposure to elevated lipids—such as 20-hydroxyeicosatetraenoic acid, a lipid metabolite derived from arachidonic acid—can act through autocrine activation of FFAR1 (GPR40), mimicking glucose-induced overstimulation of β-cells [24]. This may contribute to hyperinsulinemia and progressive β-cell dysfunction over time [26,27]. These findings support the concept that pancreatic lipid accumulation, particularly under sustained nutrient excess, may play an active role in the development of type 2 diabetes, acting alongside muscle and liver lipid overload in a broader network of ectopic lipid-induced dysfunction.

## 3. Ballooning Effect When Targeting Single Metabolic Disease

### 3.1. Obesity and Adipose Tissue Lipolysis

As discussed in Section 2.2, adipose tissue functions as the primary physiological buffer for excess energy, safely storing lipids and preventing their deposition in non-adipose tissues. However, when adipose expandability is compromised—whether by genetic defects, pharmacologic interventions, or extreme energy restriction—lipids are redirected toward skeletal muscle and liver, where they accumulate and disrupt insulin signaling [4]. This compensatory redistribution underlies what we refer to as the “ballooning effect” in obesity treatment (Figure 1A,B).

This paradox is well-demonstrated in studies that aimed to reduce adiposity as a means of improving metabolic health. For instance, the landmark work by Spiegelman’s group demonstrated that transgenic mice expressing low levels of diphtheria toxin in adipocytes displayed dramatically reduced fat mass but developed severe hypertriglyceridemia and hepatic steatosis instead of improved metabolism [28]. This foundational observation was further validated through multiple independent mouse models of generalized lipodystrophy, including the A-ZIP/F mice that express a dominant-negative protein targeting C/EBP transcription factors [29]. These “fatless” mice consistently exhibited the complete spectrum of metabolic dysfunction: diabetes, severe insulin resistance, and massive fatty liver disease [30]. The severity of metabolic complications in these models was striking—AGPAT2 knockout mice, which completely lack both white and brown adipose tissue, displayed extreme hyperglycemia, hyperinsulinemia, and hepatic steatosis so severe that liver triglyceride concentrations increased 6.4-fold in males and 2.1-fold in females compared to controls [30].

These phenotypes were largely reversed upon restoration of functional adipose tissue—either via adipose transplantation or leptin replacement therapy—highlighting the indispensable role of adipose tissue in maintaining systemic metabolic homeostasis [29]. Surgical transplantation of adipose tissue in A-ZIP/F mice and leptin supplementation in lipodystrophic mouse models both dramatically improved insulin resistance and hepatic steatosis [29]. Clinical studies in patients with congenital generalized lipodystrophy further confirm that re-establishing adipose endocrine function can dramatically improve glycemic control and reduce hepatic steatosis, despite minimal changes in body weight or caloric intake [31].

From a mechanistic perspective, many obesity therapies work by stimulating adipose tissue lipolysis to mobilize stored triglycerides. However, in sedentary or metabolically inflexible states, increased FFA release is not matched by oxidation and instead contributes to ectopic lipid accumulation in the liver and muscle [2,19]. Animal models with impaired adipose expansion, such as Pref-1 overexpression or adipose tissue-specific SPTLC2 deletion, show severe insulin resistance and hepatic lipid overload, despite having reduced fat mass [32,33]. The excess FFAs are diverted to non-adipose tissues, promoting lipotoxicity and inflammation, which further impair insulin signaling and exacerbate NAFLD and type 2 diabetes.

These findings illustrate the ballooning effect in action, whereby reducing fat mass without maintaining adipose function shifts metabolic burden rather than resolving it. Therefore, therapeutic strategies should go beyond fat mass reduction and instead aim to preserve or restore adipose tissue’s capacity for dynamic expansion and endocrine regulation.

### 3.2. Dyslipidemia, NAFLD, and Hepatic Lipid Export

Dyslipidemia has long been prioritized due to its direct link to acute, life-threatening events such as atherosclerosis and cardiovascular disease. In contrast, hepatic fat accumulation was once viewed as a slower, less critical issue. However, NAFLD is now recognized as a serious condition in its own right, with potential progression to steatohepatitis, cirrhosis, and liver cancer [34]. These two conditions share a common pathogenic axis centered on hepatic lipid trafficking. Therapies targeting hepatic lipid export to improve circulating lipid profiles may inadvertently increase intrahepatic lipid accumulation, and vice versa. In some cases, such interventions result in a redistribution of metabolic stress that exemplifies the hepatic form of the ballooning effect (Figure 1C,D). This dual burden underscores the need to consider systemic lipid handling and hormonal context when designing therapeutic strategies for dyslipidemia and fatty liver disease.

One classic example involves pharmacologic inhibition of hepatic lipid export, particularly targeting microsomal triglyceride transfer protein (MTP) and ApoB, which are essential for VLDL assembly and secretion [34,35]. MTP inhibitors such as BMS-201038 were developed to reduce VLDL production and thereby lower plasma triglyceride levels in patients with familial hypercholesterolemia [36]. Although effective in reducing LDL cholesterol and ApoB, these inhibitors led to elevated hepatic aminotransferases and hepatic fat accumulation, limiting their clinical utility due to safety concerns [34,37]. Similarly, antisense oligonucleotides targeting ApoB, such as mipomersen, improved plasma lipid profiles but increased hepatic steatosis and transaminase levels in clinical trials [38].

Further insights into the complexity of hepatic lipid handling come from studies using apolipoprotein C3 (ApoC3) transgenic mice [39,40]. ApoC3 impairs clearance of chylomicron remnants, resulting in marked hypertriglyceridemia, often exceeding 1000 mg/dL [39]. When fed a standard chow diet, these mice exhibit extreme hyperlipidemia without hepatic steatosis, due to sustained VLDL export from the liver [39]. However, under high-fat diet feeding, hyperinsulinemia suppresses VLDL secretion, redirecting hepatic lipid flux toward storage and exacerbating hepatic steatosis and insulin resistance [39]. This shift illustrates how systemic hormonal context, particularly insulin action, modulates the fate of hepatic lipids. Moreover, in patients with diabetes and severe hypertriglyceridemia, insulin deficiency may allow continued hepatic lipid export, thereby preventing steatosis despite extreme lipid load. Consistently, treatment with the MTP inhibitor lomitapide in familial chylomicronemia syndrome resulted in only mild and reversible hepatic steatosis, even with marked reductions in plasma triglycerides [41]. These patients lacked significant hyperinsulinemia, thereby maintaining VLDL export. Together, these findings suggest that the hepatic ballooning effect depends not only on reduced lipid export but also on systemic insulin levels. In hyperinsulinemic states, as in high-fat diet-fed ApoC3 mice, VLDL secretion is suppressed, promoting hepatic lipid accumulation. Conversely, in insulin-deficient conditions, such as in the lomitapide-treated patients with pancreatitis, hepatic steatosis remains mild despite reduced export. These findings underscore the ballooning effect is context-dependent and modulated by hormonal environment, and they highlight the importance of considering both lipid trafficking (input-output balance) and systemic hormonal context when designing therapies. Understanding of this hepatic “ballooning effect” is essential to develop safe and effective treatments for patients burdened by both dyslipidemia and fatty liver disease.

## 4. Strategies Toward True Disposal of Excess Lipids: Beyond Redistribution

### 4.1. Decreasing Dietary Lipid Absorption

As discussed in the preceding sections, many current therapeutic approaches merely redistribute lipids from one tissue to another, often resulting in the unintended consequence of ectopic lipid accumulation—the so-called ballooning effect. To address this fundamental limitation, more upstream strategies are required that prevent lipid overload at its source. Rather than shifting excess lipids between organs, these approaches aim for true lipid disposal by either blocking lipid entry into the body or enhancing complete oxidation before storage becomes necessary.

In this context, lifestyle interventions such as caloric restriction and exercise represent the most direct and physiological means of lowering lipid input and increasing oxidation. However, their effectiveness is often limited by poor adherence, variable cardiometabolic responses, and adverse effects such as gallstone formation and psychological distress during chronic restriction [42,43,44]. These limitations emphasize the need for novel, sustainable strategies that can modulate systemic lipid burden with fewer side effects.

One such approach is the inhibition of intestinal lipid absorption. Orlistat, a lipase inhibitor, reduces triglyceride hydrolysis and intestinal uptake but is associated with common gastrointestinal side effects, including oily stools and fecal urgency [45]. This reflects a conceptual limitation: orlistat does not dispose of lipids, but merely blocks their digestion, leaving unabsorbed fats to accumulate in the gut lumen [46].

**Figure 2:**
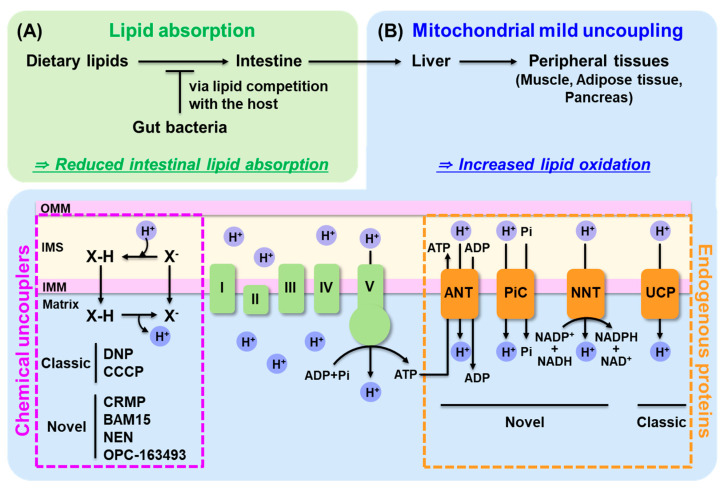
Two fundamental strategies for true lipid disposal to prevent excess lipid accumulation. This illustrates two strategies designed to eliminate excess lipids rather than redistribute them, thereby preventing ballooning effects across organs. (**A**) Inhibition of intestinal lipid absorption through gut bacteria. Gut microbes can act as a metabolic sink by directly competing with the host for dietary lipids, thereby reducing lipid uptake from the intestine. Certain bacterial strains, such as *Lactobacillus*, can incorporate or metabolize dietary fatty acids in the gut lumen, lowering host lipid absorption without increasing fecal excretion. (**B**) Mitochondrial uncoupling promotes lipid oxidation by dissipating the proton gradient across the inner mitochondrial membrane, converting nutrient-derived energy into heat instead of ATP. This process enhances energy expenditure and fatty acid oxidation in peripheral tissues, such as liver, muscle, and adipose tissue. Uncoupling can be achieved pharmacologically using chemical uncouplers—either classical protonophores (e.g., DNP, CCCP, FCCP) or safer novel agents (e.g., CRMP, BAM15, NEN, OPC-163493)—or through endogenous proteins including UCPs and recently identified transporters (e.g., ANT, PiC, NNT), which exhibit mild uncoupling activity while maintaining ATP homeostasis. Together, these approaches aim to reduce lipid overload and ectopic lipid storage, addressing metabolic dysfunction at its root. OMM, outer mitochondrial membrane; IMS, intermembrane space; IMM, inner mitochondrial membrane; DNP, 2,4-dinitrophenol; CCCP, carbonyl cyanide m-chlorophenyl hydrazine; FCCP, carbonyl cyanide p-trifluoromethoxyphenylhydrazone; CRMP, controlled-release mitochondrial protonophore; BAM15, (2-fluorophenyl)(6-[(2-fluorophenyl)amino](1,2,5-oxadiazolo[3,4-e]pyrazin-5-yl))amine; NEN, niclosamide ethanolamine; ADP, adenosine diphosphate; ATP, adenosine triphosphate; ANT, adenine nucleotide translocase; PiC, phosphate carrier; NNT, nicotinamide nucleotide transhydrogenase; NADP, nicotinamide adenine dinucleotide phosphate; NADH, nicotinamide adenine dinucleotide hydrogen; NADPH, nicotinamide adenine dinucleotide phosphate hydrogen; NAD, nicotinamide adenine dinucleotide; UCP, uncoupling protein.

A promising alternative strategy involves leveraging the gut microbiota as an active lipid sink [47]. As illustrated in Figure 2A, intestinal lipid disposal through microbial lipid competition serves as a complementary strategy that limits lipid entry. The human gut harbors approximately 38 trillion microbial cells that can compete with the host for dietary nutrients [48]. This massive microbial biomass represents a significant metabolic force that actively competes with the host for dietary nutrients and energy [49,50]. Supporting this concept, Jang et al. (2019) demonstrated that inoculating mice with specific Lactobacillus strains consume certain fatty acids, and significantly reduced intestinal fatty acid absorption and protected against diet-induced hepatic steatosis without increasing fecal lipid excretion, suggesting microbial uptake rather than passive malabsorption [51]. This finding challenges the traditional view that gut microbes only aid nutrient absorption and raises the possibility that certain probiotics may actively consume lipids within the intestine. For instance, *Lactobacillus* species have been shown to incorporate oleic acid into their membranes and convert it into cyclopropane fatty acids, enhancing their survival in acidic environments [52,53,54]. These metabolic traits suggest that probiotic strains could be repurposed as intestinal lipid consumers. Furthermore, this concept could be tested clinically: combining lipid-consuming probiotics with orlistat may reduce steatorrhea by diverting excess luminal lipids into microbial biomass. Such approaches represent a novel class of host–microbe metabolic competition strategies. Advances in lipid tracers and microbial lipidomics will enable more precise quantification of lipid flux between host and microbiota in future studies.

### 4.2. Enhance Lipid Oxidation: Mitochondrial Uncoupling

#### 4.2.1. Classic and Novel Mitochondrial Uncouplers

Mitochondrial uncoupling offers a fundamentally different strategy from lipid redistribution by facilitating true energy disposal. Unlike interventions that merely shift metabolic burden across organs, uncoupling dissipates substrate-derived energy as heat through proton leakage across the inner mitochondrial membrane, bypassing ATP synthase and reducing ATP yield [55,56]. This mechanism not only promotes lipid oxidation and energy expenditure but also reduces mitochondrial reactive oxygen species (ROS) and enhances mitophagy, thereby improving mitochondrial quality and systemic metabolic health [57,58].

Classical mitochondrial uncouplers are typically weak lipophilic acids that shuttle protons across the inner mitochondrial membrane [56]. These include 2,4-dinitrophenol (DNP), carbonyl cyanide m-chlorophenyl hydrazone (CCCP), Carbonyl cyanide-p-trifluoromethoxyphenylhydrazone (FCCP), pentachlorophenol, hydrazones, dicoumarol, salicylic acid and benzimidazole derivatives [56]. Among these, DNP gained early attention in the 1930s as an anti-obesity agent due to its ability to increase metabolic rate. However, it was withdrawn from clinical use because of serious side effects, including hyperthermia, hepatic failure, and death [59]. Despite these risks, interest in DNP has persisted due to its potent lipid-lowering properties. In preclinical models, even at low doses, DNP improved hepatic steatosis, lowered fasting glucose and insulin levels, and reduced triacylglycerol content in liver, plasma, and skeletal muscle in high-fat-fed rats [60]. These findings underscore the therapeutic potential of mitochondrial uncoupling.

To retain the metabolic benefits of DNP while minimizing systemic toxicity, researchers have developed modified analogs with liver-targeted chemical modifications or improved pharmacokinetics. Perry et al. initially developed a liver-targeted DNP prodrug by adding methyl ether moiety, which demonstrated efficacy in reversing NAFLD and insulin resistance with improved safety [61]. More recently, the same group developed a controlled-release mitochondrial protonophore (CRMP), which showed significant therapeutic benefits in both rodents and nonhuman primates with diet-induced dyslipidemia and hepatic steatosis [62,63]. CRMP improved insulin sensitivity and lipid profiles over a 6-week period without inducing hepatotoxicity or other systemic side effects. Importantly, CRMP’s efficacy was attributed to a favorable pharmacokinetic profile: it increased the area under the curve while reducing peak plasma concentrations, thus widening the therapeutic window. Toxicity thresholds were 25-fold higher than liver-targeted DNP and 1250-fold higher than standard DNP. Even at doses up to 125 mg/kg, CRMP did not elevate liver enzymes (ALT/AST) or markers of kidney injury (BUN, creatinine) [62].

Interestingly, although not chemically engineered for liver targeting, CRMP preferentially acted in the liver [62]. This liver-specific effect appears to result from its controlled-release formulation, which enables sustained low-level systemic delivery. The liver, receiving a substantial portion of portal blood flow, is therefore likely to be exposed to sufficient concentrations of the compound to promote mitochondrial uncoupling while minimizing off-target effects. Other liver-targeted DNP [61] and another analogs such as MP201, a DNP prodrug with low systemic exposure, have shown neuroprotective effects in preclinical models of multiple sclerosis [64] and Parkinson’s disease models [65], suggesting broader therapeutic applications beyond metabolic disease.

One major advancement in the field was the development of (2-fluorophenyl)6-(2-fluorophenyl) aminoamine (BAM15), a next-generation uncoupler with mitochondrial specificity. BAM15 increases proton permeability of the inner mitochondrial membrane without depolarizing the plasma membrane or inhibiting complex I [66,67,68]. BAM15 increases energy expenditure and mitochondrial respiration while preserving cellular ATP at tolerable levels. In obese mice, BAM15 decreased hepatic steatosis, reduced proinflammatory lipid intermediates, improved insulin sensitivity (confirmed via hyperinsulinemic-euglycemic clamps), and preserved lean mass and core body temperature [66,68]. The compound decreases hepatic fat, reduces inflammatory lipids, and demonstrates strong antioxidant effects [68].

Another repurposed agent, niclosamide ethanolamine (NEN), originally an anti-helminthic drug, acts as a mild mitochondrial uncoupler. NEN increases AMP-activated protein kinase (AMPK) activity by decreasing the ATP/ADP ratio, thereby enhancing fatty acid oxidation and inhibiting lipogenesis [69]. It shows liver-specific distribution with rapid metabolism (half-life ~1.25 h), contributing to its safety profile [69]. NEN reverses hepatic steatosis and insulin resistance in diet-induced obese and db/db mice [69], and has also been studied in type 1 diabetes mice [70] and in colorectal cancer model for potential roles in suppressing hepatic metastasis [71].

OPC-163493, another mitochondrial uncoupler under development, displays preferential uptake in the liver and kidneys, reducing systemic exposure [72]. It improves glycemic control independently of insulin signaling and ameliorates hepatic steatosis in preclinical models [72]. Similarly, MitoFluo, a mitochondria-targeted conjugate, also demonstrate potent uncoupling at submicromolar levels despite weak bilayer activity, implying facilitation by protein interactions [56].

Collectively, these findings support the therapeutic potential of uncoupling strategies that are tissue-specific, dose-controlled, and metabolically effective. As a true disposal strategy, uncoupling offers a promising pathway to reverse ectopic lipid accumulation and energy overload at the mitochondrial level—without merely shifting the problem between tissues.

#### 4.2.2. Novel Endogenous Uncoupling Proteins

Endogenous uncoupling mechanisms provide a physiologically integrated alternative to pharmacological mitochondrial uncouplers. In addition to the well-characterized uncoupling proteins (UCPs) (UCP1 in adipose tissues [73], UCP2 in ubiquitous tissues [74], UCP3 in skeletal muscle and brown fat [75,76], UCP4 and UCP5 (BMCP1) in brain [77]), several other inner mitochondrial membrane proteins also contribute to proton conductance and metabolic regulation.

One such family is the adenine nucleotide translocases (ANTs), which primarily exchanges ADP and ATP across the inner mitochondrial membrane. However, ANT proteins also exhibit fatty acid–stimulated proton conductance, which accounts for a substantial portion of the basal proton leak in mitochondria [78]. In addition, ANT is recognized as a key component of the mitochondrial permeability transition pore (mPTP), where its conformation is modulated by calcium overload, ROS, and changes in membrane potential [79,80]. Under these conditions, mPTP opening may allow proton flow independent of ATP synthase, contributing to uncoupling and energy collapse. These features link ANT not only to basal proton leak but also to pathological uncoupling mechanisms. Genetic ablation of ANT1 or ANT2 in mouse mitochondria halves proton leak [81,82], while overexpression in Drosophila increases it independently of nucleotide exchange [83]. ANT1-deficient animals are insulin sensitive, glucose-tolerant, and resistant to high fat diet-induced toxicity [81]. Increased sensitivity to FA-induced uncoupling and insulin sensitivity in endurance-trained male athletes is associated with ANT1 activity [84]. Beyond bioenergetics, ANT2 plays a role in metabolic inflammation. It promotes proinflammatory macrophage activation in obesity [85], which can lead to adipose tissue lipolysis and ectopic lipid accumulation [1,2,3]. ANT2 knockdown improves insulin sensitivity and reduces adipocyte hypoxia [86] and Genetic deletion of ANT2 protects diet-induced liver steatosis and insulin resistance.

Another key contributor is the mitochondrial phosphate carrier (PiC), encoded by SLC25A3. While PiC primarily imports phosphate for oxidative phosphorylation, it can also mediate H^+^/Pi symport, contributing to basal proton leak under specific conditions [87]. PiC plays an important role in negatively regulating NLRP3 inflammasome activation [88], which contributes to the chronic inflammation in obesity and insulin resistance [89]. Slc25a3 has been shown to be significantly decreased in abundance during a type 1 diabetic insult [90]. Clinically, children with a homozygous mutation in the Slc25a3 gene display mitochondrial myopathies, presenting with lactic acidosis, muscle weakness, and cardiomyopathy [91].

Nicotinamide nucleotide transhydrogenase (NNT) is another mitochondrial protein with indirect uncoupling potential. NNT couples hydride transfer between NAD(H) and NADP+ to proton translocation across the membrane [92]. Under physiological conditions, NNT uses the proton motive force to drive the reduction of NADP+ by NADH, generating NADPH for biosynthetic reactions and antioxidant defense [92]. Structurally, NNT comprises three domains: a membrane-embedded proton-channel domain flanked by two soluble nucleotide-binding domains [92]. Cryo-EM studies reveal that rotation of the NADP(H)-binding domain alternately gates the proton channel and facilitates hydride transfer, coupling redox chemistry to proton flux [92]. Functionally, NNT maintains mitochondrial redox homeostasis by supplying NADPH to the thioredoxin-peroxiredoxin system, enabling H_2_O_2_ detoxification in a respiration-dependent manner. Inhibition or genetic deficiency of NNT decreases mitochondrial NADPH levels, impairs H_2_O_2_ removal, increases ROS susceptibility, and disrupts oxidative phosphorylation efficiency [93]. Notably, a recent study demonstrated that reverse-mode Nnt is the dominant source for ROS production in heart failure, thus C57BL6/J mice lacking functional NNT were protected from the oxidative stress and death [94]. Elamipretide (SS-31) targets the inner mitochondrial membrane, interacting with cardiolipin to stabilize membranes and optimize electron transport chain function, rescued mortality induced by the reverse mode of NNT [94]. While the reverse-mode activity of NNT is pathologic, its contribution to physiological proton conductance in forward-mode remains an open question.

As discussed in Section 4.2.1 and Section 4.2.2, both pharmacological and endogenous mitochondrial uncoupling strategies offer promising avenues for true lipid disposal by promoting energy dissipation at the mitochondrial level. Chemical uncouplers provide rapid and tunable enhancement of lipid oxidation, while endogenous proteins such as UCPs, ANT, PiC, and NNT offer physiologically regulated and tissue-integrated proton conductance. Together, these complementary mechanisms can reduce ectopic lipid accumulation without provoking compensatory redistribution. Figure 2B conceptually illustrates this unified mechanism of mitochondrial uncoupling, showing proton conductance across the inner mitochondrial membrane mediated by both synthetic protonophores and endogenous proteins.

#### 4.2.3. From Traditional to Mild Uncoupling for Safety and Beyond

The concept of “mild uncoupling,” initially proposed by Skulachev [95] and Starkov [96], represents a critical evolution in mitochondrial therapeutics. Unlike classical uncouplers such as DNP or FCCP, which induce strong proton leaks often accompanied by toxicity, mild uncoupling aims to safely dissipate excess energy while preserving essential mitochondrial function [97,98]. By inducing a controlled proton leak, this strategy enhances energy expenditure and reduces reactive oxygen species (ROS), without compromising ATP synthesis [99,100]. These properties support not only metabolic improvements, but also potential benefits in muscle aging [101] and longevity [102]. The core advantage of mild uncoupling lies in its ability to trigger beneficial stress response pathways, including AMPK activation and mitochondrial biogenesis, without provoking energy collapse or cytotoxicity [103]. In contrast, classical uncouplers show narrow therapeutic windows and systemic side effects at pharmacologically active doses [58]. Considering that these are highly active compounds, it is difficult to achieve the right dose to sustain mild uncoupling without causing mitochondrial respiratory chain inhibition—a dose-sensitive risk that reinforces the importance of pharmacokinetic control, organ specificity, and conditional activation in the development of uncoupling-based therapies.

Emerging compounds like CRMP and BAM15 exemplify this safer profile. As discussed previously, CRMP exhibits liver-specific activity with an expanded safety margin in both rodent and nonhuman primate models [62,63], while BAM15 avoids plasma membrane depolarization and maintains mitochondrial specificity across a wide dose range [66]. Because organs such as the brain and heart are particularly sensitive to changes in mitochondrial membrane potential, targeting metabolically active but less vulnerable tissues like the liver and adipose tissue may represent a safer and more practical strategy for uncoupling-based therapies. These advances reflect a shift toward organ-targeted, controllable uncoupling with translational potential for chronic metabolic diseases. Complementing synthetic compounds, endogenous uncoupling systems (UCPs, ANT, and NNT) offer additional avenues for achieving tissue-specific and physiologically tuned uncoupling aligned with the body’s metabolic needs.

However, even mild uncoupling must be applied with precision. During states of high energy demand—such as intense exercise, limited oxygen supply, infection or organ stress—excessive proton leak could impair ATP availability, potentially compromising tissue function [104,105]. Moreover, even weak uncouplers have been shown to sensitize mitochondria to calcium-induced mPTP opening [106]—a high-conductance event closely linked to mitochondrial dysfunction and cell death [107]. These risks further underscore the need for uncoupling agents with tightly controllable activity within a narrow therapeutic window. Achieving this will require innovative formulation approaches, such as controlled-release kinetics, or metabolically gated activation, that enable precise dose titration and feedback-responsive modulation of uncoupling intensity under diverse physiological and pathological conditions.

Taken together, mild mitochondrial uncoupling represents a transformative strategy to reduce ectopic lipid burden and restore energy balance by promoting true metabolic disposal. As research progresses, the integration of mild uncoupling into multi-targeted therapeutic frameworks may overcome the limitations of current treatments and offer a safer, root-cause-oriented solution for complex metabolic disorders. Thus, mild uncoupling—whether achieved by synthetic agents or modulation of endogenous proteins—may form the foundation of future multi-organ metabolic interventions aimed at achieving sustainable lipid clearance and metabolic health.

## 5. Conclusions

Ectopic lipid accumulation is a common pathological driver of obesity, insulin resistance, dyslipidemia, and NAFLD. Interventions that target a single metabolic tissue or pathway often trigger compensatory lipid redistribution to other organs, resulting in new metabolic complications—a phenomenon described in this review as the ballooning effect. These outcomes highlight the need for integrated strategies that achieve true lipid disposal rather than transient redistribution.

Among emerging approaches, mild mitochondrial uncoupling offers a direct and physiologically grounded means of resolving energy surplus. By converting excess substrate energy into heat, mild uncoupling reduces ectopic lipid burden while enhancing mitochondrial quality control and metabolic flexibility. Such strategies, particularly when tissue-specific and well-tolerated, have the potential to form the basis of next-generation therapies aimed at restoring durable metabolic balance.
